# A Case of Gunshot Wound of the Intestine—Recovery

**Published:** 1854-03

**Authors:** John Neill

**Affiliations:** Surgeon to the Pennsylvania Hospital


*A Case of Gunshot Wound of the Intestine—Recovery.* By JOHN NEILL, M. D., Surgeon to the Pennsylvania Hospital.

From the Notes of Dr. JAMES DARRACH, House Surgeon.

Instances of gunshot wounds of the intestines, in which the ball was passed per anum, are so rarely reported, that Hennen, Guthrie, South in his translation of Chelius, and other writers on the subject, refer to the same case, that of Sergeant Matthews, who not only passed a ball but also a portion of the waistband of his breeches. The following case is somewhat similar.

Michael Kelly, aged 14, of sanguine temperament and good constitution, was received in the Hospital July 30th, 1853, at 5½ o'clock, P. M., with a wound of the abdomen on the left side, about two inches above the anterior superior spinous process of the ilium. He stated that he was playing with a pistol loaded with four or five gravel stones, and in attempting to cock it, it was discharged and the contents received into the abdomen. The muzzle of the fire-arm was about two inches from the abdomen at the time of the discharge. The accident happened at Bristol, twenty miles from the city, at 12 o'clock of the day of his admission. The wound was dressed by a physician of that place, and a gravel stone was removed from the wound by him. The wound was two inches long and half an inch wide, much burned and blackened by the explosion; and filling up the orifice of the wound, was a small black knuckle of intestine. The different layers of the wall of the abdomen were separated from each other around the wound, and from between the peritoneum and fascia transversalis, I removed a gravel stone about the size of a pea. Pulse 90, skin warm and moist, tongue natural, no anxiety of countenance, or restlessness, complains of no pain on pressure about the wound, tendency to drowsiness, face slightly flushed. Edges of the wound supported by adhesive straps, dressed with wet lint. The body and thighs approximated. Ordered hydrarg. chlorid. mit. ¼ gr., opii. gr. ¼ every hour. An enema of tinct. opii gtts. xl., every two hours until patient sleeps.

*Ten o'clock, P. M.*—Pulse 100, skin bathed in perspiration, face much flushed, not sleeping yet. Repeated the injection of tinct. opii gtts. xl., ordered calomel ¼ gr. pulv. ipec. opii gr. iv. every hour during the night, and the injection of tinct. opii gtts. xl. every two hours until patient sleeps. No nourishment but a tablespoonful of iced barley water every half hour.

*July* 31*st.*—Slept about three hours last night. Pulse 90, soft and full, skin warm and moist, face not much flushed, complains of slight pain about the wound, which was not dressed. Treatment continued. 7 *o'clock*, *P. M.*—Same condition, and is freely narcotized; bladder paralyzed, used the catheter. Opiates suspended.

*August* 1*st.*—Slept well all night, feels much better, has no pain. Pulse 100, soft and full, skin warm and moist, face slightly flushed, tongue coated with white fur and moist. Renewed the Dover's powder and calomel. 7 *o'clock*, *P. M.*—Pulse 97, and small, skin natural, tongue coated with white fur and moist, complains of no pain, slight tympanites, slight nausea, bladder still paralysed, used the catheter. Suspended the Dover's powder and ordered calomel gr. ¼, opium ¼, every two hours, and enema of tinct. opii gtts. xl. at bed time. Hot fomentations to the abdomen.

*August* 2*d.*—Did not rest well last night, slept about two hours. Pulse 100, and small, skin warm and moist, tongue white, furred and moist, no pain or tympanites, slightly ptyalised. He passed during the night five grumous and bloody discharges, one containing a gravel stone about the size of a large pea, which was the first evidence of a wound of his bowel. Suspended his calomel and ordered tinct. opii. gtts. x. every two hours, unless he sleeps; hot fomentation to the abdomen, barley water as diet. Passed his urine last night and this morning without assistance. 7 *o'clock*, *P. M.*—Pulse 88, full and soft, skin and tongue natural. Surface of abdomen concave, no pain in the belly on pressure. Patient says he feels very well; ordered an enema of tinct. opii gtts. xl. at bed time.

*August* 3*d.*—Pulse 84, full and soft, skin and tongue natural, no pain or tympanites, slept well during the last night. Bowels moved this morning, the discharge grumous and contained another gravel stone. Dressed the wound for the first time. The slough beginning to separate, some pain around the edges of the wound and effusion of fibrin about it. Dressed the wound with wet lint and adhesive strips, ordered tinct. opii. gtts. x. every two hours. Hot fomentation to abdomen and barley water diet. 7 *o'clock, P. M.*—Same condition as this morning, ordered an enema of tinct. opii gtts. xl. bed time.

*August 4th.*—Slept well all last night. Pulse 82, full and soft, tongue and skin natural, no pain or tympanites, local effusion of fibrine around the wound, extending to a diameter of four inches. Dressed the wound with wet lint; slough entirely separated. Suspended tinct. opii, ordered simply barley water diet. 6 *o'clock, P. M.*—Same condition, ordered enema of tinct. opii gtts. xl. at bed time.

*August 7th.*—Has continued to improve. The discharge from the wound has been sanious until to-day. The pus is now more laudable, and a gravel stone of the same size as the two passed per anum was found in the discharge from the wound. The enemata of laudanum discontinued.

After this time, the wound gradually filled up and contracted, his bowels were naturally moved, and he was discharged perfectly well on September 19th.

*Remarks.*—It will be observed that the treatment in the above case was not in conformity with the principles usually laid down by authorities upon this subject. Hennen and Guthrie recommend repeated bleedings ad deliquium animi, and consider the loss of blood, both general and local, as one of the main reliances of their treatment. In the treatment of the above case, opium was the main dependance; the object held in view was *complete rest of the bowels* by overcoming peristaltic action, and this was accomplished satisfactorily by laudanum enemata.

One of the great difficulties occurring constantly in this class of injuries, is the nausea and vomiting, therefore care should be taken not to distress the stomach of the patient with remedies, and perhaps we might even say that nothing should enter the stomach but a little cool mucilage, which is absorbed before it enters the small intestine, thus preventing peristaltic movements.

We do not of course mean to intimate that every wound of the abdomen or intestine is to be treated by laudanum injections and barley water, or that there are no cases requiring both local and general bleeding, but would merely call in question, the propriety of those teachings which inculcate the necessity of bleeding in advance, and constantly bleeding to fainting for every febrile reaction. We must expect inflammation, and *we wish its essential phenomenon, exudation*— exudation of a fibrinous kind : This is what nature attempts when she glues the intestines together, so that peristaltic motion cannot take place, and we always hope to find the convolutions agglutinated, at least in the vicinity of the wound. We do not wish a mere watery effusion, which repeated bleedings are always apt to produce, but a rich fibrinous plasma. Inflammatory symptoms are not to be feared, provided the system can stand the shock of the reaction.

The shock of the reaction as well as the shock of the injury may kill the patient, but agglutination of the bowels even if it were general, cannot in itself produce death ; it is not the effusion which kills, but the shock of the inflammatory reaction, and how can this be better obviated than by opiates ?

Another point to which we would call attention is the administration of calomel ; there are reasons why it should be given sometimes ; it may assist in preventing unnecessary intensity of inflammation, and may be useful in diminishing local congestion of the bowel by its relieving the portal circulation ; and by increasing the secretions of bile, &c., we may remove irritating ingesta. But then, on the contrary, how often does it do harm in these and analogous cases ; it unquestionably produces nausea and vomiting, unless given in very small doses and combined with opium ; it defibrinates the blood ; and the chance of having the intestines firmly glued, and the opening well-covered by a thick firm plasma is certainly diminished; and it may purge, and thus increase the peristaltic movement of the bowels, which most of all should be avoided.

It might be said in opposition that in many acute inflammations, e. g. iritis, the most decided advantages are obtained from frequent and free bleedings in conjunction with the especial effects of calomel; and this is unquestionably true. But in iritis, and in a wounded bowel or peritoneum, two very different results are to be anticipated. In the one, we *fear* that a fibrinous effusion will block up the pupil and agglutinate the fibres of the iris, and by bleeding and calomel we hope to diminish the amount of effusion, and thus render it as thin as possible in order that it may be easily absorbed. In the other, we *hope* for a fibrinous effusion, and wish the bowels to be attached.

Equal benefit may be derived from this treatment after the operation for hernia. Calomel should be especially avoided, and everything else that will increase the peristaltic movement of the bowel; instead of giving a laxative, after operation, give an anodyne; instead of a dose of oil, give opium and a laudanum enema. Instead of irritating by a purge a bowel already injured by strangulation, place it at complete rest, and let it recover from the shock already sustained. The retention of the fæces can do no harm to the bowel above the strangulated knuckle, which may be injured by disturbance. In many instances recently, after operating for hernia, we have pursued this plan with most satisfactory results.

